# How Can Speech and Language Therapists be Supported to Make Decisions Regarding Communication Therapy for People With Dementia? A Realist Theory‐Development Study

**DOI:** 10.1111/1460-6984.70346

**Published:** 2026-10-21

**Authors:** Anna Hockley, Melania Calestani, Sarah Griffiths, Suzanne Beeke

**Affiliations:** ^1^ Department of Human Communication Sciences University College London London UK; ^2^ Department of Midwifery, School of Education, Midwifery and Social Work Kingston University Kingston upon Thames UK; ^3^ Research Department of Primary Care and Population Health University College London London UK

**Keywords:** dementia, focus group, peer support, psychological intervention, psychology, social relation, staffing, stakeholder engagement

## Abstract

**Introduction:**

As of 2021, there were 57 million people with dementia worldwide, and there are 885,000 people with dementia in the UK. Communication difficulties occur in all forms of dementia. Increasing difficulty communicating thoughts and needs affects social interaction and personal relationships, with implications for individuals’ and their relatives’ quality of life. Speech and language therapists are trained to provide communication support to people experiencing communication difficulties. However, whether people with dementia are offered communication support from speech and language therapy services varies significantly across the UK. This study aimed to explain how speech and language therapists currently make clinical decisions about offering communication support, and to explain how further guidance such as a decision‐aid for clinicians may help people with dementia to receive more equitable care.

**Methods:**

This study used realist methods to develop a programme theory relating to speech and language therapist decision‐making for dementia communication interventions, in order to inform future work to develop a decision‐aid for non‐dementia specialist speech and language therapists. Theories were produced and refined through an iterative process including literature review, stakeholder engagement and focus groups with speech and language therapists to help explain how speech and language therapists make decisions and how a decision‐aid could support this process. Data was analysed through a top‐down approach using an analytic appraisal journal, and a bottom‐up approach using deductive and inductive coding.

**Results:**

Eighteen speech and language therapists participated in four focus groups. Eight theory statements were identified relating to how speech and language therapists currently make decisions about how, when and what type of communication therapy to offer people with dementia. Speech and language therapists make decisions about provision of communication therapy based on factors including staffing levels, prioritisation criteria, number of referrals received, personal beliefs, confidence, self‐perceived skills, and team culture. Eight theories relating to how a proposed decision‐aid might function were identified: provide therapy examples, peer support, service model examples, tailor for underserved groups, highlight the need for ongoing support, empower junior clinicians, and promote awareness of communication in dementia.

**Conclusion:**

This study explored how and why speech and language therapists make particular decisions when working with people with dementia experiencing communication difficulties, identifying possible mechanisms through which a decision‐aid could assist this process. A decision‐aid could address barriers to decision‐making for communication support in dementia through enhancing speech and language therapists’ knowledge, skills and confidence, while promoting equitable and sustained care.

**WHAT THIS PAPER ADDS:**

*What is already known on this subject*

- Progressive communication difficulties experienced as part of dementia can affect social interaction, personal relationships and quality of life of the person with dementia and their relatives. Speech and language therapists are well‐placed to provide communication support, but provision of this in dementia varies widely across the UK. It is unclear how speech and language therapists currently make decisions about offering communication support in dementia.

*What this study adds to the existing knowledge*

- This study uses realist methods to better understand the current decision‐making processes of speech and language therapists relating to communication support in dementia. It uses literature review, stakeholder engagement and focus groups with clinicians to develop theories to help explain how decisions are currently made, and to propose mechanisms through which a decision‐aid may help speech and language therapists to make informed decisions.

*What are the clinical implications of this study?*

- The findings suggest that speech and language therapists face significant challenges in delivering communication therapy to people with dementia due to systemic under‐resourcing, limited awareness of their role, and competing clinical priorities. The proposed decision‐aid could address some of these barriers by enhancing speech and language therapists’ knowledge, skills and confidence, while promoting equitable and sustained care. If effectively implemented, it has the potential to expand access to communication support, foster professional growth, and drive a broader recognition of communication as a vital element of dementia care.

## 1 Background

As of 2021, there were 57 million people with dementia worldwide (World Health Organization 2026). There are 885,000 people with dementia in the United Kingdom (UK), and this is set to rise (Wittenberg et al. 2019). Dementia is defined by the ICD‐10 (2019) as “a syndrome—usually of a chronic or progressive nature [that] affects memory, thinking, orientation, comprehension, calculation, learning capacity, language, and judgement” (F00‐F99). Alzheimer's Disease is the most common type of dementia (62%), followed by vascular (17%) and mixed dementia (10%) (Alzheimer's Society, 2014). Communication difficulties occur in many forms of dementia (Volkmer et al. 2024), with some rarer types such as primary progressive aphasia being primarily associated with language impairment. Dementia‐related communication impairments can include: difficulty with naming, verbal fluency, written and auditory comprehension; motor speech impairments; and cognitive communication difficulties (Gallée et al. 2025). Worsening communication difficulties in dementia are clinically relevant, correlating with reduction in employment and leisure activities, and increase in behavioural and psychological symptoms of dementia, for example, agitation, aggression and anxiety (Bourgeois et al. 2003; Schwam and Xu 2010). Increasing difficulty communicating thoughts and needs affects social interaction and personal relationships, with implications for individuals’ and their relatives’ quality of life (Braun et al. 2010; Volkmer et al. 2023). Societal perceptions of dementia may limit the participation of people with dementia in conversations and can lead to avoidance of communication (Nguyen and Lee 2020; Milne 2010).

In the UK, dementia‐specific organisations recommend the involvement of a speech and language therapist (SLT) in the management of communication difficulties (Royal College of Psychiatrists 2024) and national clinical guidance recommends that carers receive “training to help them adapt their communication styles to improve interactions with the person living with dementia” (National Institute of Clinical Excellence 2018, 46). SLTs are uniquely trained to provide this, given their expertise. The recently updated Royal College of Speech and Language Therapists’ (RCSLT) dementia position paper (RCSLT, 2024) states that speech and language therapy should be available for people with dementia and their families, highlighting the present inequity in services, with many only providing swallowing support.

There is evidence that functional communication support for dementia, which maintains communication skills using alternative communication techniques and supporting or educating conversation partners, improves target‐word use (Fried‐Oken et al. 2015), communication skills (Troche et al. 2019), and quality of life (Haberstroh et al. 2011). However, provision of support varies widely across the UK due to a variety of service models, referral criteria, individual patient needs and SLT perspectives on their role in dementia care (Hockley et al. 2025). Current service inequities compound the marginalisation of particular groups of people with dementia. Clare et al. (2025) reported that people living alone with dementia were less likely to access formal services, despite reporting social isolation and loneliness. Ethically minoritised people are underrepresented in dementia services (Parveen et al. 2017). As a result of service inequities, people with dementia and their families are not consistently offered appropriate communication support.

Some SLTs work in specialist dementia roles and have developed a wealth of clinical experience in treating dementia‐related communication difficulties. However, there are many more SLTs who encounter people with dementia on an intermittent basis, for example on the caseload of a general community therapy team. Hockley et al. 2025) survey of UK SLT service provision for people with dementia found only 24% of SLT respondents worked in specialist dementia services, the remaining 76% encountered people with dementia as part of a non‐dementia‐specialist role. Specialist dementia SLT services will not have capacity to support all referrals as the number of people with dementia continues to increase. Non‐specialist SLT services need ways to manage referrals, determining which people with dementia will benefit from what intervention at what time point. With dementia being a relatively recent addition to the SLT graduate curriculum (Volkmer et al. 2025), some SLTs may not feel fully equipped to work with this population. Therefore, clear and practical guidance is needed to support non‐dementia specialist to make these decisions.

However, clinical decision‐making is complex, and evidence suggests SLTs dynamically use a combination of intuitive and science‐informed decision‐making when making intervention choices (Durgungoz and Emerson 2021). Non‐pharmacological dementia interventions such as speech and language therapy are complex in terms of the intervention characteristics and the contexts in which they are delivered (Bartels et al. 2024). This makes it difficult to predict what outcomes will be achieved through prescribed interventions (Skivington et al. 2024) and means decision‐making is not linear. Decision‐aids have been demonstrated to successfully facilitative positive change in SLTs’ clinical practice (Jayes et al. 2020) and to support professional and patient decision‐making in dementia (Davies et al. 2019). Further insight is needed into how SLTs choose and tailor communication interventions for dementia, the contextual factors influencing their decisions, and the rationale behind them.

This study was the first step in a larger study that aims to develop and user‐test a communication‐support decision‐aid.

### 1.1 Aims

- To develop a programme theory that explains the ways in which community SLTs currently make decisions in their clinical contexts about provision of communication support to people with dementia
- To develop a programme theory to explain how a communication‐support decision‐aid could help decision‐making in the context of clinical practice.

## 2 Methods

### 2.1 Study Design

This study used realist methods to develop a programme theory relating to SLT decision‐making for dementia communication interventions and is reported according to the RAMESES II reporting standards for realist evaluation (Greenhalgh et al. 2015). Realism sits between the positivist and constructivist paradigms, positing that although a ‘real’ world exists, our understanding of reality is only partial and can be refined as new information is obtained (Davies et al. 2024). Realist methods were chosen because both SLT decision‐making and dementia healthcare provision are complex, situated in varying contexts and with individual perspectives influencing people's actions and responses to interventions.

Realist methods are increasingly adopted in complex health services evaluation, where interventions have multiple components, and outcomes are dependent on individual responses and the wider context (Wong et al. 2017a). They attempt to understand not only whether an intervention works, but to understand why it works, how, for whom, in what context and to what extent (Pawson and Tilley 2004). Realist methods can be used to review evidence and develop and evaluate interventions (Fletcher et al. 2016).

Realist research seeks to identify Mechanisms (M) by which Outcomes (O) could be achieved (or not) due to their action in different Contexts (C), see Appendix 1 for definitions of these terms. These are presented as Context‐Mechanism‐Outcome configurations (CMOcs) which are theories that seek to explain the causal processes by which an intervention works. CMOcs are often presented as ‘if…then’ statements, more clearly demonstrating their causal nature, for example, “IF the Dementia Support Worker sets up meetings with/makes referrals to/signposts to healthcare, social care and community resources AND offers support with new encounters (mechanism resources) THEN the person with dementia and carer will feel more hopeful, more confident in their own abilities, allowed to do things for themselves (carers), and will start to try out new behaviours. (mechanism responses)”. (Griffiths et al. 2025). CMOcs are developed, refined and tested, resulting in a programme theory which attempts to explain the functioning of a particular intervention. Initial programme theories are tentative and can be tested in later project stages to produce elaborated or consolidated theory. Outcomes included in theory statements are not guaranteed, they are part of a process of hypothesising potential results of particular mechanisms in particular contexts.

Realist research uses retroductive thinking, involving both inductive and deductive logic, as well as insights or ‘hunches’. Retroductive thinking “involves thinking through what causal powers might be at work in producing observed patterns” (Wong et al. 2017b, 1), with the assumption that causation cannot be understood solely through observable evidence, but requires researchers to use their knowledge, experience‐based hunches and expertise to build and test theories. Theories are developed in this way, then tested through iterative data collection and analysis to support, develop or refute them.

Realist approaches are gaining traction in speech and language therapy research due to their explanatory power. For example, Redgate et al. (2022) used realist methods to understand youth offending service delivery requirements for young people with speech, language and communication needs. Realist methods have been successfully used in dementia, for example to develop and evaluate a complex intervention for people with dementia and their carers (Griffiths et al. 2022).

### 2.2 Data Collection

Data was collected and analysed in four steps following the iterative realist methods process described by Pawson and Tilley (2004) (see Figure 1 and Appendix 2), to develop a final programme theory.

**FIGURE 1:**
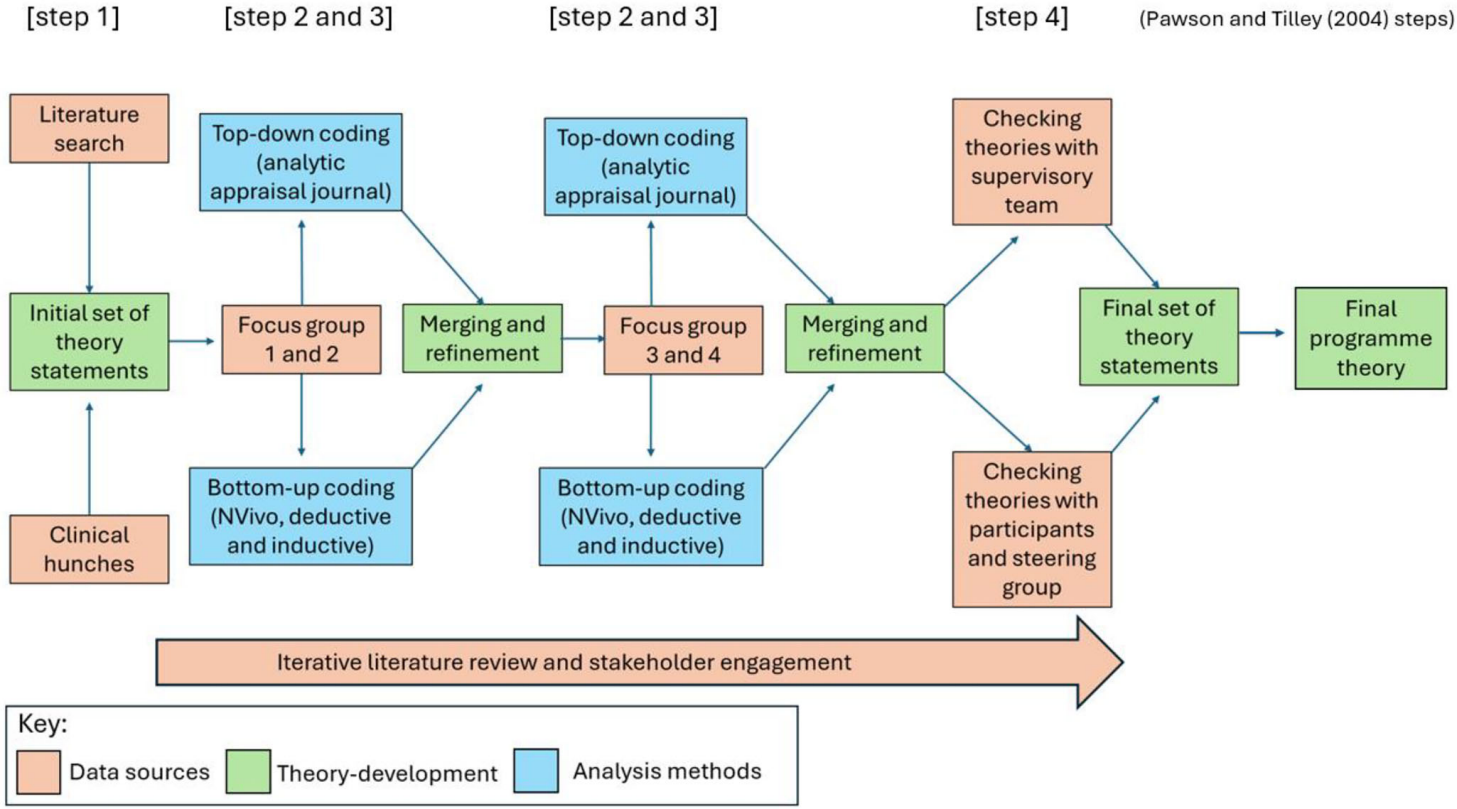
Theory‐development process. Visualisation of the steps involved in developing and refining theories.

### 2.3 Step 1: Literature Review, Insights and Clinical Hunches

The first step involved the first author, AH, developing initial theory statements (CMOcs) through background literature searching, discussion with supervisors, and stakeholder meetings to identify clinical hunches. Background literature searching is conducted within realist evaluation as one part of theory development. It is combined with the use of other sources for example, stakeholder consultation, to develop theories about lesser‐known topics, which will then be tested (Jagosh et al. 2022). The literature search employed a snowballing technique: initially identifying UK clinical guidelines about speech and language therapy in dementia, and international published research literature on clinical decision‐making by SLTs. Through this process further relevant published and unpublished literature was identified, for example, papers and blogs around SLTs’ evidence‐based practice.

Within this literature, formal, or substantive theories relevant to the topic of clinical decision‐making were identified. Formal theories are those that operate across different domains or disciplines and help explain the relationships and patterns we observe in the world, for example, attachment theory in human development (Wong et al. 2017c). In realist research formal theories “provide a bridge to a wealth of existing research and knowledge about a topic, which can inform […] building programme theory, shaping questions for evaluations, interpreting findings” (Wong et al. 2017c, 2).

In addition, AH had discussions with co‐authors MC, SB and SG and external stakeholders, this included:

- Face‐to‐face meetings with two existing dementia support groups for people with dementia and family members (September 2024 and April 2025), where the general topic of communication in dementia was discussed with an emphasis on understanding people's lived experiences.
- An online meeting with an RCSLT policy adviser (October 2024) to discuss service and policy level issues related to communication support in dementia and decision‐aid implementation strategies.
- Two online study steering group meetings (March 2023 and January 2025) including dementia specialist SLTs, an Age UK dementia adviser, and community SLTs to gather initial perspectives on areas to discuss in the step 2 focus groups, and gather perspectives on developing theories.

The early discussions led to the identification of clinical hunches, which integrated the clinical and professional experience of stakeholders and the researcher. Hunches were included in an initial programme theory along with information from the literature to undergo testing and refinement.

Step 1 generated 30 CMOcs (see Appendix 3 for examples). Following realist methods, these CMOcs are not direct quotes from sources but are a result of the researcher's retroductive thinking about causality in response to step 1 source material (Wong et al. 2017b).

### 2.4 Step 2: Focus Groups With SLTs

Focus groups with SLTs were used to refine initial CMOcs and identify new ones, enabling exploration of how decision‐making mechanisms vary across clinical contexts and individual circumstances. A focus group method was chosen because it promotes discussions, disagreements and contradictions, resulting in collection of rich data that lends itself to causal thinking (Manzano 2022). Ethical approval was gained from the University College London Department of Language and Cognition Research Ethics Committee (reference LCD‐2023‐03).

A topic guide was developed through discussions between AH, MC and SB, and included a sub‐set of CMOcs from step 1 (Appendix 4) felt to be directly relevant to the focus group topic and relevant for the SLT participants. Including CMOcs for discussion within focus groups is a feature of realist research, where theories are presented to the group for discussion. Presenting the developing CMOcs stimulates participants to give reasons for their agreement or disagreement, providing evidence from their own experiences (Manzano 2022). If topics arose that were not linked to existing CMOcs, questions were added to the topic guide for subsequent focus groups, to uncover causal insights and refine new CMOcs.

20–30 UK NHS community‐based non‐dementia specialist SLTs were sought to participate in online focus groups. Purposive sampling was used to identify a geographical spread of urban and rural SLTs across the UK, to achieve a range of contextual information. Recruitment was via the RCSLT's research newsletter, Clinical Excellence Network email lists, the researchers’ professional contacts, and X (formerly Twitter). Participants completed an online consent form and provided anonymous demographic information prior to participating in the focus group. Four focus groups were conducted by AH online via Microsoft Teams with between 2 and 7 participants each, Groups 1 and 2 were conducted between May and June 2023 with a total of 13 participants. These considered the CMOcs developed from the step 1 literature search and generated new CMOcs from participants’ experiences. Groups 3 and 4 were conducted between November‐December 2025 with a total of 5 participants. Groups 3 and 4 used an updated topic guide with developing CMOcs from groups 1 and 2, and additional open questions targeting key areas lacking an existing CMOc. In this way, groups 3 and 4 permitted final refinements to the developing CMOcs and provided new perspectives on key areas without developed theory. An SLT PhD student co‐facilitator was present to co‐facilitate groups 1 and 2. Focus groups lasted between 1 h 21 min and 1 h 25 min, were video recorded and automatically transcribed using the MS Teams record function, with the researcher checking, correcting and anonymising the transcription manually after each group.

### 2.5 Data Analysis

Influenced by the realist analysis method of Maben et al. (2024), the focus group data was analysed in two ways; top‐down using an analytic appraisal journal, and bottom‐up using deductive and inductive coding on NVivo 14 (Lumivero 2023). These methods were employed simultaneously as a means of triangulation and to allow for broader causal insights to be identified in top‐down analysis, and more detailed nuances in the bottom‐up approach. Once both were completed, AH checked the developing set of CMOcs against the analytic appraisal journal and the list of NVivo codes and their definitions to ensure no key insights were missed, and that the CMOcs provided a logical and explanatory summary of the focus group data.

### 2.6 Top‐Down Analysis

For the analytic appraisal journal, the researcher followed the process described by Maben et al. (2024). This involves reading and analysing each focus group transcript, then, using a free‐write approach, summarising the key insights from the transcript in a reflexive manner following the headings: big idea; what surprised me; key learnings; to explore further. SB, MC and SG then read the transcript and the journal entry, adding their own comments and insights.

### 2.7 Bottom‐Up Analysis

The bottom‐up process followed the steps described by Gilmore et al. (2019) and described by Griffiths et al. (2025). The initial CMOcs from the informal literature search were collected on MS Excel. Once focus groups 1 and 2 were complete, these initial CMOcs were transferred to NVivo, with a node created for each CMOc. The anonymised transcripts for focus groups 1 and 2 were uploaded and read multiple times to identify causal insights. Data supporting original CMOcs was coded under the existing CMOcs, and new causal insights led to the creation of inductive nodes. Subsequent related data was coded to those, or child‐nodes created for related but more specific detail (see Figure 2 for examples). Once focus group 1 and 2 coding was complete, the researcher re‐read the analytic appraisal journal, and the coding on NVivo, merging any related codes and adding new CMOcs or adjusting the original CMOcs. This set of refined CMOcs was then taken to focus groups 3 and 4, and the steps above followed again to analyse that data.

**FIGURE 2:**
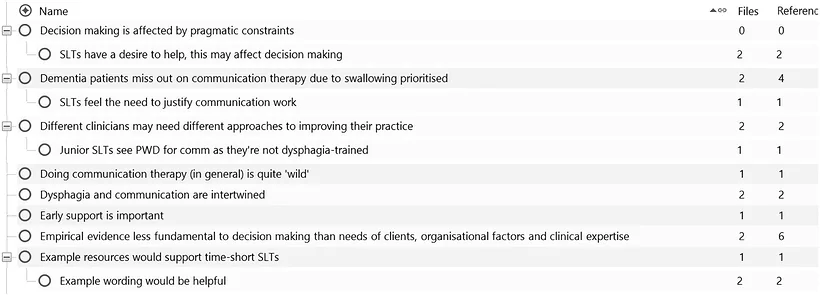
Nvivo coding example. Lists a selection of codes and sub‐codes created on nvivo during the analysis process.

### 2.8 Step 3: Refining Theories

The above process resulted in a set of 25 CMOcs which were discussed by the study authors, at which point CMOcs which related to the same or similar causal processes were merged and some re‐worded for clarity. A subset was reviewed by the study's steering group—including a dementia specialist SLT, a community SLT, and an Age UK representative—who provided experiential feedback, leading to minor wording refinements for clarity.

The final set of condensed CMOcs was emailed to focus group participants for member‐checking. Three responded, offering brief feedback on the statements and sharing additional experience‐based reflections.

## 3 Rigour

Reflexivity strengthens theory development in realist research by acknowledging the impact of personal values (Downey et al. 2024). The researcher maintained a reflexive journal throughout data collection and analysis, using it to track the evolution of each CMOc in an audit trail and examine subjectivity. For example, reflecting on how the researcher's initial view of a ‘fair’ service being one which is the same as other similar services was challenged by finding out more about how services needed to differ in order to respond to local need. These reflections influenced the questions asked in focus groups and CMOc development.

The researcher participated in a multidisciplinary realist support group that met twice during data analysis to review the developing CMOcs. Their feedback helped identify potential biases, improve clarity for broader audiences, and ensure CMOcs were explanatory.

Data for the study was collected through multiple data sources (literature review, focus groups, supervisor review and stakeholder engagement), enhancing credibility through data triangulation (Noble and Heale 2019).

## 4 Results

### 4.1 Participant Demographics

Eighteen SLTs participated in the focus groups. Table 1 presents demographic information by focus group and in total, as certain details—such as years of experience—are relevant to findings from specific groups (e.g., theory about junior SLTs emerged from focus group 4).

**TABLE 1:** ‐ Focus group participant demographics. Documents for each demographic how many participants fell into that category in each focus group and in total.

Demographic	Category	Focus group 1 (*n* = 6)	Focus group 2 (*n* = 7)	Focus group 3 (*n* = 3)	Focus group 4 (*n* = 2)	Total (*n* = 18)
		No. (%)	No. (%)	No. (%)	No. (%)	No. (%)
Years post qualification	Newly qualified	0	1 (14%)	0	0	**1 (6%)**
	1–2	2 (33%)	1 (14%)	1 (33%)	2 (100%)	**6 (33%)**
	3–4	1 (17%)	0	0	0	**1 (6%)**
	4–10	2 (33%)	2 (29%)	0	0	**4 (22%)**
	10+	1 (17%)	3 (43%)	2 (67%)	0	**6 (33%)**
NHS banding	5	2 (33%)	1 (14%)	1 (33%)	0	**4 (22%)**
	6	2 (33%)	4 (57%)	1 (33%)	2 (100%)	**9 (50%)**
	7	2 (33%)	1 (14%)	0	0	**3 (17%)**
	8	0	1 (14%)	1 (33%)	0	**2 (11%)**
Geographic location	London	1 (17%)	0	2 (67%)	0	**3 (17%)**
	Scotland	1 (17%)	1 (14%)	0	0	**2 (11%)**
	South East England	1 (17%)	0	0	1 (50%)	**2 (11%)**
	South West England	1 (17%)	0	0	0	**1 (6%)**
	Yorkshire and The Humber	1 (17%)	0	0	0	**1 (6%)**
	Wales	1 (17%)	2 (29%)	0	0	**3 (17%)**
	East Midlands	0	3 (43%)	1 (33%)	0	**4 (22%)**
	West Midlands	0	1 (14%)	0	0	**1 (6%)**
	Northern Ireland	0	0	0	1 (50%)	**1 (6%)**
Clinical setting	Community	5 (83%)	5 (71%)	3 (100%)	2 (100%)	**15 (83%)**
	Mixed (community and inpatient/rehab)	1 (17%)	2 (29%)	0	0	**3 (17%)**
Service able to see people with dementia?	Yes	4 (67%)	6 (86%)	3 (33%)	1 (50%)	**14 (78%)**
	Limited by dementia type	1 (17%)	0	0	1 (50%)	**2 (11%)**
	Limited (e.g., one‐off session only)	1 (17%)	1 (14%)	0	0	**2 (11%)**
How many patients with dementia you have personally seen in the past 24 months (for communication or swallowing)	< 50	2 (33%)	3 (43%)	2 (67%)	1 (50%)	**8 (44%)**
	50–100	0	1 (14%)	0	0	**1 (6%)**
	101–500	2 (33%)	3 (43%)	1 (33%)	1 (50%)	**7 (39%)**
	501+	2 (33%)	0	0	0	**2 (11%)**

SLTs ranged from newly qualified to those with more than 10 years’ experience. Participants spanned the NHS Agenda for Change bandings and were from across the UK. Most were based solely in community settings (*n* = 15, 83%), with three (17%) in mixed community and inpatient roles. Whilst most participants’ services allowed them to see all people with dementia for communication support (*n* = 14, 78%), four services (22%) were limited either by dementia sub‐type or were able to provide one‐off communication sessions only. Participants estimated the number of people with dementia they had seen for speech and language therapy over the past two years using range options. Responses varied widely: 8 (44%) saw fewer than 50, 7 (39%) saw 101–500, and 2 (11%) saw over 500. This reflects the unequal service provision for people with dementia and demonstrates how some SLTs have substantial clinical experience and others limited exposure.

### 4.2 Theory‐Statements

CMOcs pertained to both the current decision‐making of SLTs (8 CMOcs, Appendix 5), and also the potential ways a decision‐aid could support them (8 CMOcs, Appendix 6). Each will be discussed in turn.

### 4.3 How Do SLTs Currently Make Decisions?

Eight CMOcs were identified relating to how SLTs currently make decisions about communication therapy for people with dementia (Appendix 5). This describes the context in which a decision‐aid would need to function, with this set of CMOcs informing the contextual elements of the final programme theory (below). The data revealed that SLTs make decisions about provision of communication therapy based on factors including staffing levels, prioritisation criteria, number of referrals received, personal beliefs, confidence, client need, self‐perceived skills, and team culture. A decision‐aid should recognise the pressured, understaffed settings many SLTs work in, while aligning with their beliefs about communication therapy and fostering development of skill and confidence. Three examples will be presented here, with evidence examples that informed their development.

### 4.4 CMOcA1: Workplace Culture Combined with Existing Clinical Interests Influences Provision of Communication Intervention

***If communication therapy for people with dementia is not commissioned, but staffing levels are adequate and SLTs have a clinical interest in dementia (Context), and they are supported by management to practise independent clinical decision‐making (Mechanism—resource), then SLTs have more confidence in their abilities and are more likely to use their own clinical judgement to treat a person with dementia for communication rather than just dysphagia (Mechanism—response), resulting in people with dementia receiving clinically appropriate treatment (Outcome)***.

SLTs used their clinical judgement to determine whether to override service criteria restrictions, based on perceived client need:

“We aren't funded to provide a communication service for adults with dementia. So, we kind of use our own discretion […] we just sort of tried to use a bit of clinical judgment to look at that specific person in terms of the aims of the referral, what the goal is.” (P07)

Management oversight affected these decisions: for some, lack of oversight enabled independent clinical decision‐making based on client need:

“Because the post was kind of new and when I started, there wasn't really anyone looking at me and checking what I was doing, I've kind of done my own thing […] I got a few referrals for primary progressive aphasia and I've been finding my way through that, which I still feel like I have no idea what I'm doing there despite trying very hard to learn” (P01)

SLTs’ personal feelings about communication support influenced their decisions:

We weren't commissioned to see people with dementia for more than […] one assessment session. Personally, because I felt very passionate about it, I was fairly flexible with that. And I was often seeing them for swallowing anyway, so I'd then try and give a little bit more input with other things, but it was difficult. (P17)

Member‐checking participants agreed with CMOc 1:

“I 100% agree with this! The fact that our service has a decent communication provision for patients with dementia is definitely down to individual therapists being passionate about communication difficulties in dementia, and us being lucky enough to have the support of line management to invest time into developing this side of the service”. (P02)

### 4.5 CMOcA2: Lack of Awareness of the SLT Role in Communication Affects SLT Decision‐Making

***If there is limited awareness of the SLT role in communication in dementia across an organisation and staffing levels are low/services are stretched (Context) then commissioners will press for dysphagia to be prioritised (Mechanism—resource), so SLTs will be concerned about an influx of referrals if they were to increase awareness of their role in communication therapy,  because there may be no increased recognition, funding or staffing to see people for communication input (Mechanism—response), resulting in the service not providing equal access to support for communication and dysphagia in dementia (Outcome)***.

Some participants reported that referrers were not aware of the role of speech and language therapy in dementia communication:

“It's making me realise just how few referrals I actually get for communication and dementia. The thinking seems to be so ingrained that that's just not what we do […] I mean I work in an office with memory nurses and I've had conversations where they literally say to me ‘but there's nothing you can do is there, for communication’” (P04)

In teams that did not receive referrals, participants were reluctant to attempt awareness‐raising because they were not clear on their service availability:

“If they did refer to us and then we're going to say, oh, actually, we're not commissioned and we can't really see them. So then they probably will stop referring because we're not being clear about our role and we don't feel confident in our role.” (P17)

Whilst many participants reported a lack of referrals to SLT for communication, others were overwhelmed by the numbers but did not have capacity to support them:

“Our referral numbers were such that there was no negotiation on that because the minute we started to accept them, the risk was that you would end up with an influx and we just didn't have the staffing to support it. So, we had an advice sheet and that went out as standard along with the statement just explaining that there wasn't a service in this area to support these people and we would be there for the swallowing.” (P05)

### 4.6 CMOcA6: Lack of Referrals Leads to Skill‐Loss

***If referrers to SLT have limited awareness of the role of SLT in communication in dementia (Context), then very few referrals are received (Mechanism—resource), so SLTs lose skills and confidence in treating dementia‐related communication difficulties (Mechanism—response), resulting in the SLT workforce not treating this population***.

Participants reported a vicious cycle of deskilling due to lack of funding for dementia communication support:

“For me it's really difficult because of the lack of funding, the lack of support for this, from commissioners, it's a real unmet need in our area. So, it's very, very difficult […] not able to provide the service that you want to provide is very frustrating and also you then get into that downward spiral of deskilling.” (P08)

A contributing factor was what some felt to be ‘minimal’ training and skills development in dementia communication during SLT training:

“The majority of the workforce when they had their training it wasn't really spoken about, and I'm two years post‐graduation but still it was very minimal, and most of my knowledge and interest I got on one of my placements because I was lucky enough to be with a person who had a very special interest in dementia” (P02)

Member‐checking participants agreed this was an issue:

“Agree. Also, I feel that a lot of the time the skills to manage dementia‐related communication difficulties aren't taught/explored in the first place and maybe never developed if there's no pathway in the service in which you work” (P18)

### 4.7 How Could a Decision‐aid Support Decision‐making?

Eight CMOcs relating to how a proposed decision‐aid might function were identified (Appendix 6) and are all presented here as they have important implications for decision‐aid development. These CMOcs were based primarily on participants’ suggestions for the decision‐aid but also included the researcher's inferences about discussion points that held relevance for the planned decision‐aid.

### 4.8 CMOcB1: Therapy Examples Support Decision‐Making

***Where SLTs have clinical expertise in communication therapy (Context) and SLTs are provided with examples of therapy ideas/case studies (Mechanism—resource), then they will be able to use their own expertise to make informed decisions about who would benefit most from what therapy and how, and will make decisions more confidently (Mechanism—response), resulting in a person with dementia receiving efficient and effective treatment (Outcome)***.

This was one of the most strongly supported CMOcs, as SLTs felt that they lacked knowledge of what sorts of interventions might benefit people with dementia and their families:

“But then even if I worked out […] who to see first—I would still be thinking: ‘and do what with them?’. So actually, my question might be if I knew what do to with them, I could probably suss out myself [who to see]” (P01)

“I think almost having a list of ‘which of these areas is impacting you the most at the moment?’ and actually this is like one example of a goal that we could work towards and yeah, that might seem a bit restrictive, but within that we all know that we can work in a really person‐centred way and that's very important in speech therapy”. (P12)

Participants agreed with this CMOc during member‐checking:

“I think therapy ideas/case studies being made available would be very useful and encourage SLTs to attempt treatment for people with dementia. I think therapy examples could be shared and adapted to each individual.” (P18)

A decision‐aid could provide therapy examples through case studies.

### 4.9 CMOcB2: Access to Clinical Expertise or Peer Support Helps Decision‐making

***If an SLT does not have clinical expertise in dementia (Context) and is provided with opportunities to discuss their management of communication difficulties in dementia either with specialist dementia SLTs or with their own peers, (Mechanism—resource), then SLTs feel supported in their decision‐making and more confident in their approach (Mechanism—response), resulting in SLT knowledge development and increased likelihood of people with dementia receiving communication support (Outcome)***.

For junior SLTs in teams that are not formally seeing people with dementia for communication, peer support was a valuable resource to improve confidence and knowledge in treating people with dementia:

“And we're restricted by not having it commissioned […] I've always felt like I shouldn't really be doing it […]. But then we had a bit of a breakthrough recently where during a peer support session, we realised actually a few of us are doing the same thing and then we had a journal club about it.” (P18)

Access to more specialist dementia SLTs was felt to be a potentially helpful avenue for decision‐making support, particularly around the challenging aspect of knowing when to stop therapy:

“When you keep trying lots of different things, but either they're not taking it on board or they're not practising and following through. It's then making that decision to say, OK, well, we're not making any progress. We've tried lots of things. And then it's also the experience, I suppose we're a general community service as well. So, we don't have the specialist dementia knowledge within that, so I've got a colleague in the older adult mental health service that I will ask quite a lot of questions of saying, I've tried this. It's not helping. What else could I try?” (P13)

“The kind of support groups and sharing ideas would be beneficial because my community service doesn't have any dementia specialist working in it and I wouldn't know who to ask”. (P11)

The decision‐aid could provide access to peer or specialist support through online forums or regular online peer support sessions.

### 4.10 CMOcB3: Existing Service Models Support Decision‐Making

***Where SLT services have well‐developed pathways or treatment approaches for communication intervention in dementia (Context), SLTs can ‘fit’ dementia referrals to the existing model (Mechanism—resource), feeling more able to make decisions about whether to accept a dementia communication referral (Mechanism—response), resulting in certain people with dementia receiving treatment that is suitable for them, but others potentially receiving generic or non‐individualised care (Outcome)***.

Some SLTs reported their services had existing pathways for supporting people with dementia who were experiencing communication difficulties. For example, one participant reported that dementia communication referrals were usually considered for a dementia communication group:

“You make a decision based on, and again, because we've started our group that we're really trying to develop, so anyone that seems appropriate for the group we want to offer them a place in the group, but then again it's basically up to the therapist to decide whether we think they are appropriate.” (P02)

Other participants agreed that pre‐determined pathways were helpful:

“I agree, we have a pathway for all dementia referrals and what the service aim to offer, which has been really helpful.” (P02)

However, clinical judgement was still needed to determine whether a people with dementia was appropriate for an established type of therapy and participants expressed concern at generic pathways not suiting the person‐centred approach required in dementia care:

“The whole point of dementia therapy is that it's got to be tailored to the individual. So, if you apply formula to somebody, you're not necessarily providing equitable care”. (P10)

One participant described a shift—first in children's services and now emerging in dementia care—toward public health‐oriented, non‐referral models of communication therapy driven by SLT‐assessed need:

“Children's services […] have moved away from just the request for assistance or referral model to much more proactive interventions […] and there's a lot of talk about moving in that direction in adult services as well. So, if we're providing a sort of proactive intervention such as this communication project in care homes, it's not responding to referrals, it's much more, we've identified a general need and we can throw something at it.”

For decision‐aid development, possible care pathways or treatment approaches could be suggested, but the importance of individualised care must be recognised. The decision‐aid could provide examples of non‐referral based, public health type approaches to communication support in dementia to circumvent the issue of inconsistent referrals.

### 4.11 CMOcB4: Tailoring Support for the Most Underserved Groups Improves Equity of Care

***For the most underserved groups of people with dementia (for example, those with moderate dementia and those living alone) (Context), if a newly‐developed resource takes into account their needs in particular (Mechanism ‐resource), then clinicians will feel able to provide individualised, appropriate care to these most underserved sub‐groups (Mechanism—response), resulting in reduction of care inequity and improved clinical outcomes for the most vulnerable patients (Outcome)***.

Participants reported that there were certain groups of people with dementia who were not often referred to SLT services, for example those with moderate dementia:

“I quite often see people either right at the beginning or when they're quite severe for communication, but not much in the middle when they're in that moderate stage where they might be managing a little bit but need a bit of help, they would get a bit missed I think.” (P12)

For individuals living alone with minimal family involvement, SLTs felt less equipped to offer ongoing communication support and noted that care provision would be more complex due to challenges such as difficulty remembering appointments:

“Unfortunately, they got left a little bit if they didn't have the family to take over what you were suggesting. So, I'd say that was one of the main factors in what and how much input they got, unfortunately.” (P17)

“I think they probably do get lost in the system if they've not got someone who's living with them or someone who's seeing them on a regular basis. […] All of the patients who come to see me so far, they've either been accompanied or the one lady who came by herself, she does live with her daughter. So, I haven't even thought about patients with dementia living alone”. (P16)

The decision‐aid needs to consider different groups of people with dementia and how SLTs can tailor their approaches to communication support so as not to exclude underserved groups.

### 4.12 CMOcB5: A ‘Middle Ground’ Resource Supports Implementation of Guidance

***Where national dementia guidance is, by necessity, general, and shared therapy resources are individualised (Context), if a newly‐developed resource situates itself in the middle ground, providing case studies alongside general learning points (Mechanism—resource), resource‐users will relate these to their own clinical experiences, feeling supported to trial communication therapies with people with dementia (Mechanism—response), resulting in more people with dementia receiving effective and efficient communication support and improving their wellbeing (Outcome)***.

Participants debated their preferences for generic versus detailed guidance. Some felt that a new decision‐making resource must not be too specific as this could jeopardise person‐centred care, which they considered very important in dementia, whereas others felt that generic guidance was not helpful, practically.

“I sit on the fence as usual and I think that it depends on partly your amount of experience in that field. So I think there has to be some specific guidance there, but you have to have the flexibility to abandon it and use your own judgment.” (P10)

One participant suggested that it was possible for guidelines to be both detailed and flexible, through suggesting approaches to try but acknowledging that all people are different:

“I think you can have incredibly detailed guidelines that you can […] get lots of ideas from and you can have the flex […] The guidelines should reflect the fact that all people are different and these are things that you could try. I would prefer guidelines [that] apply to everybody newly qualified, experienced people […] I like the ASHA dementia guidelines [they] give you ideas as well and support.” (P09)

For the decision‐aid, different levels of detail should be included to allow for both practical applicability and individual flexibility. For example, there could be the option to navigate from more general principles of communication therapy in dementia to case studies of specific interventions with detail about which people with dementia these interventions were effective/not effective for.

### 4.13 CMOcB6: Often More Support Is Needed Than a One‐Off Session Can Provide

***As a diagnosis of dementia can be difficult to adjust to, and by nature affects memory (Context), if SLTs offer only a one‐off session or an advice sheet about communication difficulties and their progression (Mechanism—resource), then there is a risk that people with dementia and their family members will feel unduly worried, lack ongoing support, and/or will not remember what has been advised (Mechanism—response), resulting in potential reduced engagement with services, poor therapy outcomes and a negative impact on the wellbeing of the person with dementia/their family (Outcome)***.

Many participants reported that people with dementia were offered one‐off communication assessment sessions or a one‐off advice sheet about communication in dementia, either due to service stipulations or management advice. This left some participants feeling uncomfortable:

“You're not quite doing a good job of providing it, but you're also not really doing what you're meant to do and not doing it at all. You're kind of like dipping. It feels a bit like maybe not very satisfying or fulfilling because you're trying, but not quite” (P17)

Some participants reported that this was not the most effective way of supporting communication in dementia:

“It just takes times the communication ones. It really does. It just takes, you know, more support.” (P18)

Participants felt that for some people with dementia and families, a one‐off advice session would be enough, but that for others this could be counter‐productive:

I've got another gentleman with Alzheimer's who I gave some clear speech strategies […] and I gave him a handout, and when I called up on the phone […] he wasn't able to recall the speech strategies. So, I offered to send him a little card in the post, and he's agreed that he's going to keep it in his pocket or in his wallet for reference so that [he] can remember more easily. I feel like if it weren't for me following up with that phone call then […]ʼʼ (P16)

SLTs are operating in stretched services, often with restrictions on the number and type of sessions they can offer. The decision‐aid should recognise this context while also considering the potential drawbacks of limiting ongoing support, particularly considering the memory deficits experienced in dementia.

### 4.14 CMOcB7: Junior SLTs Provide Potentially Unharnessed New Insights Into the Communication Needs of People With Dementia

***If communication input is largely provided by newly qualified SLTs who are still working on their dysphagia competencies (Context), then they will have fresh, insightful (albeit sometimes unrealistic) ideas for improving communication input and services for this population (Mechanism ‐resource) but may not have the confidence or experience to enact these ideas or to provide advice on this to others (Mechanism—response) resulting in missed opportunities to improve communication support services for this population (Outcome)***.

Whilst support from dementia‐specialist SLTs was useful, participants discussed how years of experience did not necessarily equal more dementia‐communication knowledge. Junior SLTs reported seeing most of the dementia communication referrals due to not being dysphagia trained, with more senior SLTs seeing only dysphagia. This led junior SLTs to build experience‐based knowledge.

ʻʻThere was a good long time where I was just a communication therapist and […] when I started as a band five was I was the only therapist really doing communication. So everyone else you know, more senior people, more experienced people were doing dysphagia […] and then the pattern tends to be then as the band fives become more confident and have more experience and expertise in communication, when their dysphagia training comes around, the same cycle happens again and they lose the confidence and they are just working in dysphagia then. (P18)

A decision‐aid should consider the possibility that experienced SLTs may see fewer people with dementia for communication support than junior SLTs, and how the experience‐based knowledge developed by junior SLTs can influence service‐development.

### 4.15 CMOcB8: Current Shifts in Perception of the Importance of Communication in Dementia Provide an Optimum Environment for a New Resource

***Where there is a whole system shift towards increasing awareness of the impact of dementia on communication among healthcare professionals, service providers and the public (Context), if a newly‐developed resource could harness this enthusiasm (Mechanism—resource), then stakeholders would feel the resource is important and meaningful to people with dementia/families (Mechanism—response), resulting in a wider reach of the resource, with implications for further increased awareness of communication difficulties, increased referral rates to SLT, and recognition by service funders (Outcome)***.

SLTs reported sometimes working in clinical teams with a defeatist culture around communication support for dementia, which more newly‐qualified SLTs were concerned by:

“It did kind of seem like, oh, what's the point? That was a kind of general vibe I got, but because I was new to speech therapy I was really quite shocked and kind of outraged by this.” (P17)

However, they felt this viewpoint may be changing:

“I think people's minds are opening a little bit to the to the idea that this can really help people. And you know, if it's not our role to do that, whose role is it really, to support people with their communication?” (P18)

A decision‐aid should seek to advocate for the importance of communication support in dementia even within the SLT profession, to clarify our role. Reference to the 2024 RCSLT Dementia Position Paper would support this, with a possible impact on referrals, as one participant mentions:

“We can't expect them [referrers] to know our role if we don't.” (P17)

### 4.16 Programme Theory

The programme theory summarises the contexts (from the first set of theory‐statements), mechanisms and outcomes (from the second set of theory‐statements) for the proposed decision‐aid (Figure 3).

**FIGURE 3:**
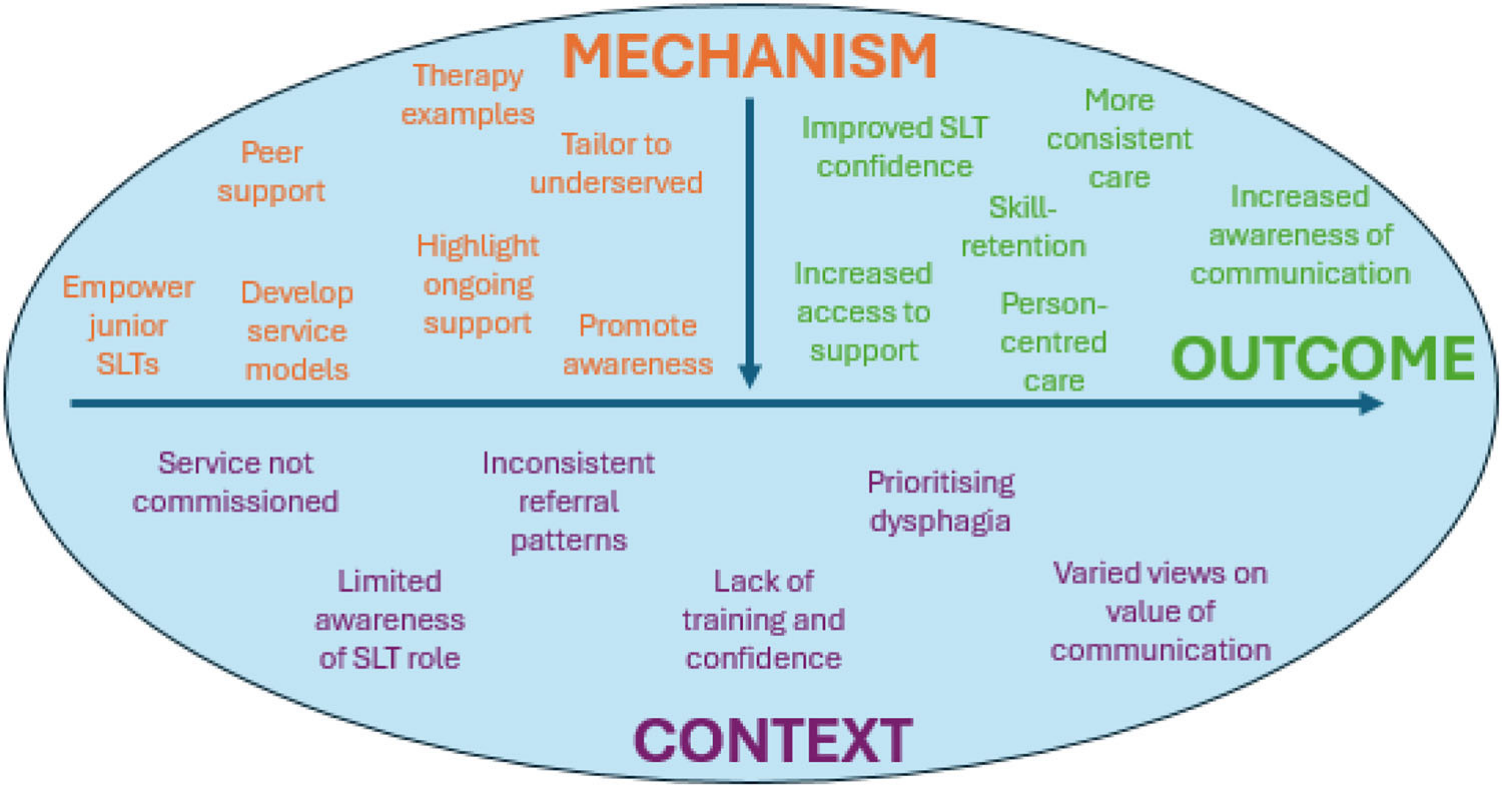
Programme theory. Visualises the contexts, mechanisms and outcomes identified in the study.

## 5 Discussion

This study used realist methods to identify how and why SLTs make particular decisions when working with people with dementia experiencing communication difficulties, to postulate theories to explain what is currently happening in practice, and how a decision‐aid could support decision‐making.

This study found numerous factors influence current SLT decision‐making for communication in dementia, including client need, service limitations, staffing levels, prioritisation criteria, and awareness. These affect the number of referrals received. Personal factors including beliefs about the importance of communication support for dementia, experience, confidence, and self‐perceived skills also contribute. Team culture was also important. Support from management to make independent decisions, and observing colleagues, influenced decisions. These factors align with McCurtin and Clifford (2015), who identified that the main influences on SLT decision‐making were practice evidence (i.e., professional experiences), and pragmatic constraints (i.e., contextual factors), with external evidence (i.e., research) playing less of a role. A decision‐aid needs to consider SLTs making choices based on their own clinical settings, colleagues’ perspectives, clinical experiences and personal values. It should use these sources of evidence, for example through providing a platform for SLTs to share case studies, service models and receive feedback to develop skills and confidence. Case studies are an underutilised way of “providing evidence about context and transferability, but also for helping strengthen causal inferences when pathways between intervention and effects are likely to be non‐linear.” (Paparini et al. 2020, 1). This is often the case for dementia communication interventions.

In the focus groups there was little discussion of the actual communication interventions SLTs currently provide to people with dementia, aside from advice sheets and one‐off advice sessions. Similarly, there was limited discussion about how decisions were influenced by aetiology, age, or severity. Participants reported receiving few referrals for dementia communication, and when they did receive referrals, often having limitations on what they were able to offer. This may have resulted in SLTs not having to think through *what* they would offer in terms of interventions, besides one‐off advice, or how their approach may differ depending on dementia sub‐type, stage or severity.

This study developed a programme theory explaining how a decision‐aid for dementia‐communication therapy could function in practice. Whilst decision‐aids have been demonstrated to support complex decision‐making (Davies et al. 2019), they may be better suited for use by patients and family members rather than clinicians, due to their longer length. However, shorter tools such as heuristics that is, rules of thumb, though in some ways more suitable for clinicians, may over‐simplify the complex nature of dementia and communication therapy (Davies et al. 2018; Davies et al. 2016; Mathew et al. 2016). SLTs in the present study reflected on the need for a decision‐aid to include suggested therapy options, concluding that these would act as a mechanism to stimulate the use of their own clinical judgement to determine whether a specific client would benefit from that intervention. Whilst some expressed concern that therapy examples or detailed guidance might limit flexibility for person‐centred care, others felt that there was a happy medium where a decision‐aid could provide detailed guidance but leave room for independent judgement. The American Speech Language Hearing Association (ASHA) Dementia Guidance (n.d.) was mentioned as a good example of detailed but flexible guidance. This guidance provides an overview of dementia‐related communication difficulties, with clickable options to more detailed, evidence‐based descriptions of, for example, therapy options, and case studies including functional goals structured within the International Classification of Functioning (World Health Organization 2001) framework to ensure a person‐centred approach. A newly developed decision‐aid could use a similar approach to structuring case studies, which could be provided by the SLT community based on their own practice experience.

Junior SLTs may have more recent and comprehensive knowledge and experience of supporting people with dementia with communication than experienced SLTs, who may take primary responsibility for dysphagia. This finding suggests that involving junior SLTs in initiatives to improve decision‐making and services for communication in dementia will ensure their key perspectives are included. Patel and Stebbings (2019) found that junior clinicians were well placed to lead new service development, reporting benefits for quality of care and staff retention. However, there may be teams in which more experienced SLTs regularly work with dementia communication, and more experienced clinicians will have service development expertise, and so all perspectives need to be included when developing dementia services Some participants in the present study reported frustration at clinical teams not prioritising communication because this did not fit with their personal values/beliefs. Enabling SLTs to lead projects to demonstrate the importance of communication‐focussed work may improve staff satisfaction with knock‐on effects for staffing levels.

Participants raised concerns about underserved sub‐groups of people with dementia including people with moderate dementia and those living alone without family support. Nationally, there is a focus on people living alone with dementia with recognition that this group is often overlooked and increasing in number (Clare et al. 2025). Dementia services have been recommended to review their approach to ensure they are inclusive and communicating with people in a way that meets their needs. Where dementia communication intervention often involves committed input from a communication partner (El‐Wahsh et al. 2021), the decision‐aid may need to include specific information about how to meet the communication needs of people living alone without regular support. Similarly, a decision‐aid could provide specific case studies of SLTs’ work with people with moderate dementia to encourage others to attempt therapy with this group.

This study has demonstrated the complexity of decision‐making in communication support for people with dementia. Whilst a decision‐aid is not able to address all the issues identified (for example, the lack of dementia communication referrals some teams report), future research could consider ways in which to address awareness and referrals. Similarly, some teams cited lack of commissioned SLT services and low staffing as key reasons for offering one‐off advice sheets instead of intensive therapy for people with dementia. A decision‐aid will not be able to address service funding and staffing directly. However, awareness and understanding of the causal influence of these contextual factors on SLTs’ decisions will permit the decision‐aid to utilise some of these influencers, for example it could facilitate SLTs to share business cases for dementia service funding and support junior SLTs to share service‐development ideas.

### 5.1 Strengths and Limitations

Information was gathered from a range of sources‐including research literature, clinical hunches, clinical guidance, stakeholders including people with dementia and their relatives, and SLT participants‐using an iterative approach to produce a programme theory. The CMOcs developed have been checked by a range of relevant parties to ensure that they adequately reflect people's experiences. Using realist methods has resulted in CMOcs that have practical applications, demonstrate connections between contexts, mechanisms and outcomes and that sit with the complexity of the real‐world rather than trying to simplify it into constituent parts.

The study's sample could have been larger—initially 20–30 SLTs were sought but only 18 participated in the focus groups. This may reflect overstretched clinical services, with SLTs juggling many competing priorities. Had there been a greater number and range of SLT participants, some of the theories developed may have been more nuanced. For example, although the present findings suggest more senior SLTs largely take responsibility for dysphagia in dementia, other perspectives may have arisen in SLT teams that share communication referrals between members with different experience levels. The study could have more formally included people with dementia and families in CMOc development for example, through focus groups or interviews with them. Realist theories are complex and can be challenging to present in an accessible way. Further work is needed to explore how people with cognitive impairment can be included in realist studies. This will support the inclusion of people with lived experience of dementia more directly in future realist research.

## 6 Conclusion

This study aimed to explain how SLTs currently make clinical decisions about offering communication therapy, and to explain how a decision‐aid for clinicians may help people with dementia to receive more equitable care. SLTs face significant challenges in delivering communication therapy to people with dementia due to systemic under‐resourcing, limited awareness of their role, and competing clinical priorities. The authors now plan to develop the proposed decision‐aid, to address some of these barriers by enhancing SLTs’ knowledge, skills and confidence, while promoting equitable and sustained care. If effectively designed and implemented, it has the potential to expand access to communication support, foster professional growth, and drive a broader recognition of communication as a vital element of dementia care.

## Funding

This study is funded by the Economic and Social Research Council.

## Ethics Statement

Ethical approval was gained from the University College London Department of Language and Cognition Research Ethics Committee (reference LCD‐2023‐03).

## Conflicts of Interest

The authors declare no conflicts of interest.

## Data Availability

The data that support the findings of this study are available from the corresponding author upon reasonable request.

## Appendix 1 Realist terminology.

### 1.1

**Table:**

Term	Explanation (adapted from RAMESES II guidelines (**Wong et al**. 2017a))
Context	A set of rules, norms, values and interrelationships, pre‐existing any intervention under development, that operate and make a difference to the outcomes for example, social, economic and political structures, intervention participants and staffing, geographical and historical context
Mechanism (resource and response)	The interaction between what the intervention provides (e.g., decision‐aid offers the resource of ‘example goals’ for SLTs to use (resource)) and the participants’ reasoning (understanding, values and beliefs (response)) that enables the intervention to work (e.g., the example goals appeal to SLTs’ beliefs that communication therapy should meaningfully support the PWD's participation)
Outcome	Intended and unintended consequences of a mechanism e.g., example goals lead to non‐individualised care (unintended) or example goals lead to meaningful goal‐setting with PWD (intended)
Context‐Mechanism‐Outcome configuration (CMOc)	A hypothesis about the relationship between context, mechanism and outcomes (Kirsh et al. 2017), e.g. “If SLTs have time and opportunity to access online resources (Context), and example goals from real‐life case studies are provided through a searchable online repository (Mechanism–resource), the goals will appeal to SLTs’ belief in meaningful communication therapy (Mechanism – response), leading to meaningful goal‐setting with PWD (Outcome)”.

## Appendix 2 Theory development steps.

### 2.1

**Table:**

Step	**Pawson and Tilley** (2004) description	Methods used in this study	
1	Develop an understanding of the intervention and develop initial programme theory	Initial programme theory developed through: thematic literature review, insights from discussion with stakeholder groups, and hunches from the researcher's clinical experiences.	Iterative literature review and stakeholder engagement throughout the data collection and analysis process
2	Collect data to test the theories and address core research questions	Focus groups with SLTs tested and refined the initial programme theory and addressed the main research questions.	
3	Analyse data to interrogate the developing programme theory	Inductive and deductive close data coding of focus group transcripts for causal insights. Analytic appraisal journaling following each focus group to uncover wider causal insights.	
4	Interpret findings to explain, revise and refine the initial programme theory and to develop a refined theory	Focus groups, stakeholder meetings, member‐checking and research team discussion facilitated further interpretation of the developing theory to produce a final programme theory.	

## Appendix 3 Examples of initial theory‐statements (CMOcs).

### 3.1

**Table:**

Theory‐statement (CMOc)	Source	Relationship to formal theory or model
If SLTs do not have a specific and uniform way to select an intervention; instead, they have dynamic decision‐making practices determined by multiple factors, then it is complex to predict what outcomes will be achieved through particular prescribed interventions	Decision Making of Speech and Language Therapists: Science or Intuition? (Durgungoz and Emerson 2021)	The study examined decision‐making through the lens of cognitive‐continuum theory (Hammond 1981)
If SLT services provide equal access to intervention for communication and swallowing disorders, then PWD's needs will be met	RCSLT dementia position paper (2014) (note the RCSLT dementia guidance had not yet been updated at the time of initial programme theory development).	None
If SLTs had more specific evidence‐based guidance on effective interventions for PWD experiencing communication difficulties, they would more regularly offer communication advice/therapy alongside a dysphagia assessment, because they would have more confidence that what they are offering would be helpful/effective.	Clinical experience and hunches	None
For more experienced clinicians, the decision‐aid could confirm the decisions they have already made (improving confidence), and for more junior therapists, the decision‐aid could act to prevent harms for example, caution against one‐size‐fits‐all approaches.	Stakeholder consultation – dementia specialist SLT	None
For PWD who struggle with reading, if SLTs provide only written information and advice, then PWD will struggle to engage or benefit from this advice.	Stakeholder consultation – person with dementia	None

## Appendix 4 Focus group topic guide.

### 4.1

What are the communication therapy decision‐making practices and needs of community speech and language therapists working with PWD?

**Welcome and introduction (5 min)**: Welcome and thank you for agreeing to take part in this focus group and for coming along today. We really appreciate you taking the time to participate as your views are really important to us.

You're participating in this group as you're a speech and language therapist working with adults in the community. Today, we're interested in finding out about your experiences working with PWD. We're particularly interested in how you make decisions about provision of communication support and therapy for this population. This will feed into the design and piloting of a clinical decision‐aid for speech and language therapists to use.

This focus group will be video and audio recorded. Once transcribed all of your names will be removed so quotes from the discussions can be used in reports but no‐one, apart from the study researchers, will know who said what. After the group, certain video clips will be selected to be used as ‘trigger films’ in the next stage of the study, to help stimulate discussion. Before doing this, I will contact you individually if a clip has you in it, to ask your permission to use it in the presentations. The videos will not be shared anywhere else and will be deleted after that. Please ask any questions about this now.

Anything that is said in today's focus group should remain confidential.

If there are any questions or discussions that you do not wish to answer or participate in, you do not have to do so; however please try to answer and be as involved as possible.

So now to just to run through some ground rules for the group:

1. In order to make a record of any important point, we ask participants to speak one at a time (raise hand function).

2. We are interested in your views, so there is no right or wrong answer.

3. You do not have to speak in any particular order.

4. If you have any questions before we start, please be confident to ask them.

Some questions will be open questions, and at other points I will present ‘theory‐statements’, which are attempts at understanding what is happening in practice that I have developed based on my own experience and reading. These will be presented in order to gauge your views on whether you agree/disagree with them and what you think is really going on.

**Table:**

START RECORDING			
Main questions	Sub questions/prompts	Theory‐statements	Timings
*Introductory questions*			
Tell us about your experience of seeing patients with dementia in your setting	How often do you see these patients? Who refers them Is it largely for communication or swallowing, or both? What does your service stipulate?	If SLT services provide equal access to intervention for communication and swallowing disorders then PWD's needs will be met If SLT services for PWD are within an integrated multidisciplinary context, then the philosophy and goals of intervention will be shared and consistent If SLTs do not see PWD for communication difficulties then referrers will be unaware of this potential avenue of support	8 min 3.10–3.18
What communication difficulties do you notice in this client group?	What do PWD and families report they are struggling with in terms of communication? What do you observe? What is the impact of these difficulties on the client or their family?		8 min 3.18–3.26

*Main questions*			
How do you assess the communication of and/or communication support needs of PWD?	Informal/formal assessment	If assessment and treatment is individualised, draws from the broad range of approaches available and takes account of the evidence, then communication skills of the person with dementia and the carer will be optimised	7 min 3.26–3.33
What type of support or therapy do you provide?	What is the content of the interventions? What interventions are you aware of, even if you haven't used them? Direct/indirect? How many sessions? Uni or multi‐disciplinary?		7 min 3.33–3.40
How do you decide whether to provide communication therapy or support?	Service‐limitations? Dependent on patient/family priorities? Individual clinicians’ decision? Timing?	therapists’ rationales for using interventions for clients appeared to be based primarily upon their perceived needs of clients (the primary stakeholders), with organizational factors and clinical expertise playing a role, with existing empirical evidence being less fundamental to decision making	7 min 3.40–3.47
How do you decide what type of communication support or therapy to provide?	Based on patient needs? Based on clinical guidance? Based on previous experience? Based on service limitations?	If clinical guidance is quite general, then SLTs have more flexibility to offer communication support that is tailored to the individual If clinical guidance is quite general, then SLTs may not know who to offer support to, how much, and when	7 min 3.47–3.54
How do you decide how long to continue communication therapy?			7 min 3.54–4.01
How do you find making decisions about provision of communication support and therapy?	Do you find it easy to decide whether or not to provide therapy and of what type? Have you experienced any difficulties making these kinds of decisions? What are the main barriers? what helps?	Difficulties knowing what communication support to provide might be affected by what kind of provision this service already has. If SLTs had more specific evidence‐based guidance on effective interventions for PWD experiencing communication difficulties, they would more regularly offer communication advice/therapy alongside a dysphagia assessment, because they would have more confidence that what they are offering would be helpful/effective.	8 min 4.01–4.09

*Ending questions*			
What would help you to make these communication‐ support and therapy decisions?	Further education/training courses? More research in the field? Accessible summaries of the sorts of therapy to offer for example, in a decision‐aid	“Attempts to improve practice should focus on a range of evidence sources and take into account clinician's specific needs depending on career stage, post‐qualification status and patient group factors of their practice.”	8 min 4.09–4.17
If a decision‐aid were designed to help with this, what would it look like?	Electronic/online? Paper? Interactive? Would it require a training course? What would make you more likely to use a tool like this? Where should the decision aid be located?	A decision aid might be most useful for those areas where SLT services are limited and could have the greatest impact there. “Involving patients in the shared decision making process is essential for achieving patient‐centred care and reaching decisions that are informed and based on individual patient values” If SLTs do not have a specific and uniform way to select an intervention; instead, they have dynamic decision making practices determined by multiple factors, then it is complex to predict what outcomes will be achieved through particular prescribed interventions.	8 min 4.17–4.25
Is there anything else you'd like to say/add?			3 min 4.28
Closing statement, thanking of participants, next steps (2 min)			
			**Total: 90 min**

## Appendix 5 CMOcs relating to current SLT decision‐making.

### 5.1

**Table:**

Category	Title	Context	Mechanism resource	Mechanism response	Outcome	Quotes	Other supporting information
**Service related**	CMOcA1. Workplace culture combined with existing clinical interests influences provision of communication intervention	If communication therapy for PWD is not commissioned, but staffing levels are adequate and SLTs have a clinical interest in dementia	And SLTs are supported by management to practise independent clinical decision‐making	then SLTs have more confidence in their abilities and are more likely to use their own clinical judgement to treat a person with dementia for communication rather than just dysphagia	resulting in PWD receiving clinically appropriate treatment	“We weren't commissioned to see PWD for more than […] one assessment session. I mean personally, because I felt very passionate about it, I was fairly flexible with that. And I was often seeing them for swallowing anyway, so I'd then try and give a little bit more input with other things, but it was difficult”. (P17)	Member‐checking: “I 100% agree with this! The fact that our service has a decent communication provision for patients with dementia is definitely down to individual therapists being passionate about communication difficulties in dementia, and us being lucky enough to have the support of line management to invest time into developing this side of the service”.
	CMOcA2.Lack of awareness of the SLT role in communication affects SLT decision‐making	If there is limited awareness of the SLT role in communication in dementia across an organisation and staffing levels are low/services are stretched	then commissioners will press for dysphagia to be prioritised	so SLTs will be concerned about an influx of referrals if they were to increase awareness of their role in communication therapy, because there may be no increased recognition, funding or staffing to see people for communication input	resulting in the service not providing equal access to support for communication and swallowing in dementia	“It's making me realise just how few referrals I actually get for communication and dementia. The thinking seems to be so ingrained that that's just not what we do […] I mean I work in an office with memory nurses and I've had conversations where they literally say to me ‘but there's nothing you can do is there, for communication’”(P04)	
	CMOcA3.Communication referrals often being allocated to junior SLTs may affect service‐development in this area	If communication input in dementia is largely provided by newly qualified SLTs (because they are still working on their dysphagia competencies)	then senior SLTs will not see these clients	so lose skills, knowledge and confidence in providing communication therapy	resulting in clinicians who have the experience and position to make service‐level changes not being able to draw on recent clinical experience in this field and potentially meaning service developments do not take into consideration the communication needs of PWD	“I think it's just again tricky not to go on about the same thing again, but being like very much just starting out and realising, oh, there's a big like deficit in the service here, but there's nothing that you feel that you can do about it because you're only starting and you're like, oh, well, that's just the way it is.” (P18)	
**Referrals**	CMOcA4.Clearly defined, shared referral criteria result in improved referral rates	For clinical teams where there is not a clear or defined pathway for communication support offerings for PWD available to referrers	if the SLT team share clear, defined criteria for referral with referrers	then referrers will have confidence in referring PWD	resulting in increased (appropriate) referral rates to SLT	“I do feel that our patients are being a little bit at the mercy of who's on triage that day and that it's quite a subjective issue in our team, which means that somebody might pick the referral up and do one thing, and without any real pathway there, the next day if they're picked up by a different member of the team the outcome might be completely different” (P05)	
	CMOcA5.Opening up to referrals for communication input in dementia risks unmanageable demand	If SLT teams are under‐staffed and facing many clinical pressures	and a team decides to accept or invite communication referrals for PWD	n/a	then there is a risk that the team will become overwhelmed with referrals and be unable to cope with the increased demands	“And then if you're opening it up and making that awareness, then we haven't got that service to actually support, if then, we were getting the same amount of communication referrals as we were dysphagia, I think we'd be even more inundated than we are now.” (P06)	Member‐checking: “Potentially true – I think this is a significant barrier to accepting communication referrals for PWD, as a lot of teams will already be under pressure”
**Skills**	CMOcA6.Lack of referrals leads to skill‐loss	If referrers to SLT, for example memory clinicians, GPs, third‐sector organisations, have limited awareness of the role of SLT in communication in dementia	then very few referrals are received	so SLTs lose skills and confidence in treating dementia‐related communication difficulties	resulting in the SLT workforce not treating this population.	“For me it's really difficult because of the lack of funding, the lack of support for this, you know from commissioners, it's a real unmet need in our area. So it's very, very difficult […] not able to provide the service that you want to provide is very frustrating and also you then get into that downward spiral of deskilling so you don't have the skills to deal with that client group and the less of it you do the less skilled you are. So you know you're getting that kind of vicious cycle.” (P08)	Member‐checking: “Agree Also I feel that a lot of the time the skills to manage dementia‐related communication difficulties aren't taught/explored in the first place and maybe never developed if there's no pathway in the service in which you work”
	CMOcA7.The deprioritisation of communication in dementia leads to skill‐loss	Where national guidance for communication intervention in dementia is, by necessity, general and communication intervention is deprioritised due to service‐level guidance prioritising dysphagia	SLTs have limited opportunities to develop clinical experience in this area	losing confidence in their knowledge of treating this client group	resulting in SLTs not treating this group and continuing to lose skills and confidence	“We are very, very short staffed between vacancies and sick leave. There's only a few of us left. So yeah, so it is a struggle. And as as P16 is saying, yes, obviously we can't prioritise communication and dementia even though for some it would be a priority, of course. But of course we, you know, with a number of referrals that we get for dysphagia and support, then we need to prioritise those referrals”. (P15)	
	CMOcA8.Supporting junior SLTs with service development skills helps develop clinically meaningful dementia communication services	If communication input is largely provided by more junior SLTs	and these junior SLTs are given training, support and opportunity to develop/improve services	then they will be able to harness their fresh perspectives, more recent evidence‐based knowledge (from their SLT training) and direct clinical knowledge of the communication needs of PWD	to develop clinically meaningful services to support the communication needs of PWD	“there's so many people who who do need to support and we could support that just don't have access to the service for whatever reasons. And yeah, part of that obviously is just awareness and awareness of us. I think there probably is work that could be done there to help with that. Just I don't know exactly what that would be.” (P18)	

## Appendix 6 CMOcs relating to design of a new decision‐aid.

### 6.1

**Table:**

Title	Context	Mechanism resource	Mechanism response	Outcome	Quotes	Other supporting information	Implications for decision‐aid
CMOcB1. Therapy examples support decision‐making	Where SLTs have clinical expertise in communication therapy (but not necessarily in communication therapy for dementia)	SLTs are provided with examples of therapy ideas/case studies with outcomes for example, through an online repository	SLTs feel encouraged to attempt communication therapy for PWD SLTs are able to combine their own expertise (practice knowledge) with case study information and knowledge of their individual patient	+ SLT can decide which PWD would benefit from SLT +PWD/their family receive efficient and effective person‐centred communication therapy ‐ Potential for PWD to receive ‘generic’ therapy if focus is too much on the example rather than the individual	“But then even if I worked out what to do—who to see first—I would still be thinking: ‘and do what with them?’. So actually, my question might be if I knew what do to with them, I could probably suss out myself [who to see]” (P01) “I think almost having a list of right, the which of these areas are you? Is impacting you the most at the moment and actually this is like one example of a goal that we could work towards and yeah, that might seem a bit restrictive, but within that we all know that we can work in a really person centered way and that's very important in speech therapy”.		Provide therapy examples through case studies
CMOcB2. Access to clinical expertise or peer support helps decision‐making	Where community SLT teams do not have specialist dementia SLTs Where communication therapy for PWD is not prioritised within the SLT or multi‐disciplinary team Where junior SLTs often provide communication support in dementia	SLTs are provided with a forum to discuss communication management for PWD, either through: ‐ Access to specialist dementia SLTs ‐ Peer‐support for example, peer supervision	SLTs feel more confident in deciding which PWD would benefit from communication support SLTs (particular junior SLTs) feel reassured that they are ‘doing the right thing’ even if it does not fit with what the rest of their team are doing	+ Improved SLT knowledge and skills in communication therapy for PWD + Increased likelihood of PWD who would benefit from communication support receiving it	“Yeah, I'm. I just wanted to say that, yes, I I do think training and research and support groups have a really, you know, it's really a really valuable contribution and it's really useful to to be able to access those. And I guess my situation is that I'm is a generalist community caseload. And I think the referrals for PWD, with communication needs are perhaps, you know. I could count them on the fingers of one hand, you know, per year. So it's hard to kind of. Justify the time out of your clinical time to train for something that's not your specialism. It's really hard. And then like I say, you get into that downward spiral of deskillling. It's all just very, it's all very difficult. I think if you don't have that specialism within your within your service.” And we're restricted by not having it commissioned that you know, technically, I've always felt like I shouldn't really be doing it, if that makes sense. It's kind of like, oh, I shouldn't really tell anyone I'm doing this, but then we had a wee bit of a breakthrough kind of recently where during like a peer support revision, we kind of realised actually a few of us are doing the same thing and then we had a journal Club about it after that and.	Member checking: “It would be good to have greater access to other SLTs specialising in this area.”	Provide opportunities for peer and expert support
CMOcB3. Existing service models support decision‐making	Where SLT services have structured pathways or treatment approaches for communication intervention in dementia for example, communication groups, pro‐active communication training in care home	SLTs can ‘fit’ dementia referrals to the existing model (for example, directing a new referral to an already established dementia communication group	SLTs feel more able to make decisions about whether to accept a dementia communication referral	+ Certain PWD receive appropriate communication support ‐ Others receive generic or non‐individualised care	“You make a decision based on, and again, because we've started our group that we're really trying to develop, so anyone that seems appropriate for the group we want to offer them a place in the group, but then again it's basically up to the therapist to decide whether we think they are appropriate” (P02)	Member‐checking: “I agree, we have a pathway for all dementia referrals and what the service aim to offer, which has been really helpful.”	Provide opportunities for developing structured care pathways or service models
CMOcB4. Tailoring support for the most underserved groups improves equity of care	For the most underserved groups of PWD (for example, those with moderate dementia and those living alone)	SLTs are provided with specific case studies/therapy examples relating to targeting these specific needs	SLTs feel encouraged to try communication therapy with these less‐encountered groups SLTs feel supported to develop services to support these groups	+ Reduction of care inequity and improved clinical outcomes for the most vulnerable patients	“And I found that, I think just thinking through listening to other people are saying I quite often see people either right at the beginning or when they're quite severe and for communication, but not much in the middle when they're kind of in that moderate stage where they might be managing a little bit but need a bit of help, they would kind of get a bit missed I think.”		Provide specific guidance for the most underserved groups
CMOcB5. A ‘middle ground’ resource supports implementation of guidance	Where national dementia guidance is by necessity quite general, and shared therapy resources are individualised	SLTs are provided with case studies alongside general learning points about communication provision for PWD	SLTs will relate case studies and learning points to their previous clinical experiences SLTs will feel supported to offer communication therapies to PWD	+ Communication therapy is offered to the right PWD at the right time + Improvement in PWD/family's wellbeing	“I sit on the fence as usual and I think that it depends on partly your amount of experience in that field. So I think there has to be some specific guidance there, but you have to have the flexibility to abandon it and use your own judgment.” (P10)		Provide case studies along with more generalisable guidance
CMOcB6. Often more support is needed than a one‐off session can provide	A diagnosis of dementia can be difficult to adjust to, and by nature affects memory	SLTs offer a one‐off session or an advice sheet about communication difficulties and their progression SLTs spend adequate time getting to know the person and family across follow‐up sessions	There is a risk that PWD and their family members will feel unduly worried, lack ongoing support, and/or will not remember what has been advised The person with dementia will feel heard. SLT will feel able to confidently offer therapy that they believe will address the goals of the individual	‐ Potential reduced engagement with services, poor therapy outcomes and a negative impact on the wellbeing of the person with dementia/their family + Increased engagement in therapy and meaningful clinical outcomes for PWD/family	“I'm yeah, I don't think currently like I'm supported to work with communication with dementia patients. They do ask me to discharge them even if I have identified, like, there's a communication need and they kind of said to me, like, you can give, like, these advice sheets or you can, like, leave I just got told I can leave parts, some activities to do with them. And that's kind of all I'm able to kind of do.” “It's actually what we're working towards with this person and that can be so general and so varied that it's hard to pin down within one session or even three or four sessions, particularly with dementia, when there's cognitive stuff going on as well”		Provide information about risks/benefits of one‐off versus more continued support
CMOcB7.Junior SLTs provide potentially unharnessed new insights into the communication needs of PWD	If communication input is largely provided by newly qualified SLTs (who are still working on their dysphagia competencies)	then they will have fresh, insightful (albeit possibly sometimes unrealistic) ideas for improving communication input and services for this population	but may not have the confidence or experience to enact these ideas or to provide advice on this to others	‐ missed opportunities to improve communication support services for this population	“There was a good long time where I was just a communication therapist and actually just my situation when I started as a band five was I was the only therapist really doing communication. So everyone else you know, more senior people, more experienced people were doing dysphagia so […] and then the pattern tends to be then as the band fives become more confident and have more experience and expertise in communication. When their dysphagia training comes around, the same cycle happens again and they lose the confidence and they are just working in dysphagia then. And that seems to be the pattern.” (P18)	Member‐checking: “Agree. Newly qualified SLTs may be less cynical and willing to try new approaches that they are aware of/have read about etc!”	Include junior SLTs in resource‐development, consider ways junior SLTs can be supported to develop services.
CMOcB8. Current shifts in perception of the importance of communication in dementia provide an optimum environment for a new resource	Where there is a whole system shift towards increasing awareness of the impact of dementia on communication among healthcare professionals, service providers and the public	A newly developed resource harnesses this enthusiasm	Stakeholders will feel the resource is important and meaningful to PWD/families	Wider reach of the resource Implications for further increased awareness of communication difficulties Increased referral rates to SLT Recognition by service funders	it did kind of seem like. Oh, what's the point? That was a kind of general vibe I got, but because, you know, I was new to speech therapy. I was really quite Shocked and kind of outraged by this.		Present resource at SLT and dementia events, engage with media discussions about dementia, use social media to support the resource to gain traction
